# Bi-Functional Alginate Oligosaccharide–Polymyxin Conjugates for Improved Treatment of Multidrug-Resistant Gram-Negative Bacterial Infections

**DOI:** 10.3390/pharmaceutics12111080

**Published:** 2020-11-11

**Authors:** Joana Stokniene, Lydia C. Powell, Olav A. Aarstad, Finn L. Aachmann, Philip D. Rye, Katja E. Hill, David W. Thomas, Elaine L. Ferguson

**Affiliations:** 1 Advanced Therapies Group, School of Dentistry, College of Biomedical and Life Sciences, Cardiff University, Heath Park, Cardiff CF14 4XY, UK; l.c.powell@swansea.ac.uk (L.C.P.); hillke1@cardiff.ac.uk (K.E.H.); thomasdw2@cardiff.ac.uk (D.W.T.); fergusonel@cardiff.ac.uk (E.L.F.); 2 Norwegian Biopolymer Laboratory (NOBIPOL), Department of Biotechnology and Food Sciences, NTNU Norwegian University of Science and Technology, 7491 Trondheim, Norway; olav.a.aarstad@ntnu.no (O.A.A.); finn.l.aachmann@ntnu.no (F.L.A.); 3 AlgiPharma AS, 1337 Sandvika, Norway; phil.rye@algipharma.com

**Keywords:** gram-negative bacteria, multidrug resistance, polymer therapeutics, colistin, polymyxin B

## Abstract

The recent emergence of resistance to colistin, an antibiotic of last resort with dose-limiting toxicity, has highlighted the need for alternative approaches to combat infection. This study aimed to generate and characterise alginate oligosaccharide (“OligoG”)–polymyxin (polymyxin B and E (colistin)) conjugates to improve the effectiveness of these antibiotics. OligoG–polymyxin conjugates (amide- or ester-linked), with molecular weights of 5200–12,800 g/mol and antibiotic loading of 6.1–12.9% *w*/*w*, were reproducibly synthesised. In vitro inflammatory cytokine production (tumour necrosis factor alpha (TNFα) ELISA) and cytotoxicity (3-(4,5-dimethylthiazol-2-yl)-2,5-diphenyltetrazolium bromide (MTT) of colistin (2.2–9.3-fold) and polymyxin B (2.9–27.2-fold) were significantly decreased by OligoG conjugation. Antimicrobial susceptibility tests (minimum inhibitory concentration (MIC), growth curves) demonstrated similar antimicrobial efficacy of ester- and amide-linked conjugates to that of the parent antibiotic but with more sustained inhibition of bacterial growth. OligoG–polymyxin conjugates exhibited improved selectivity for Gram-negative bacteria in comparison to mammalian cells (approximately 2–4-fold). Both OligoG–colistin conjugates caused significant disruption of *Pseudomonas aeruginosa* biofilm formation and induced bacterial death (confocal laser scanning microscopy). When conjugates were tested in an in vitro “time-to-kill” (TTK) model using *Acinetobacter baumannii*, only ester-linked conjugates reduced viable bacterial counts (~2-fold) after 4 h. Bi-functional OligoG–polymyxin conjugates have potential therapeutic benefits in the treatment of multidrug-resistant (MDR) Gram-negative bacterial infections, directly reducing toxicity whilst retaining antimicrobial and antibiofilm activities.

## 1. Introduction

Antimicrobial resistance (AMR) is a significantly growing global challenge that is associated with elevated morbidity and mortality rates, high healthcare costs and >700,000 deaths annually [1,2]. Excessive use of antibiotics in animal husbandry, agriculture, and human and veterinary medicine has contributed to a dramatic increase in life-threatening multi- and pan-drug resistant bacterial infections [3]. This environmental exposure has been compounded by decreases in the development of novel antimicrobials and it has been predicted that AMR could result in 10 million annual deaths by 2050 [4]. According to the World Health Organisation (WHO), Gram-negative bacteria such as carbapenem-resistant *Pseudomonas aeruginosa* and *Acinetobacter baumannii*, extended spectrum β-lactamase-producing and carbapenem-resistant *Klebsiella pneumoniae* and *Escherichia coli* represent a major clinical threat and burden to public health [5]. It has been estimated that Gram-negative bacterial resistance resulted in 960,000 hospital admission days in Europe in 2017 [6].

**Scheme 1:**
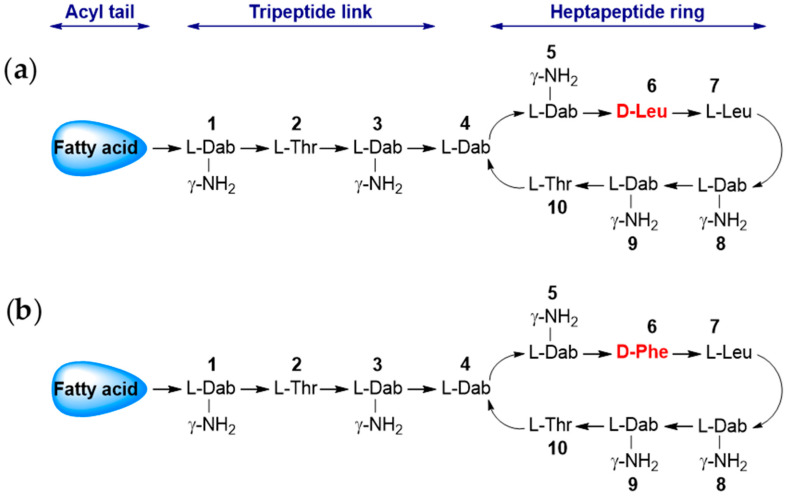
Graphic structure of (**a**) colistin and (**b**) polymyxin B. The hydrophilic heptapeptide ring is linked to a hydrophobic acyl tail through a tripeptide fragment. The only structural difference between both molecules is an amino acid residue at position 6: d-leucine in colistin is replaced by d-phenylalanine in polymyxin B. Composition of the fatty acyl tail: 6-methyloctanoic acid for polymyxin B1/E1 and 6-methylheptanoic acid for polymyxin B2/E2.

Polymyxins (Scheme 1), such as polymyxin B and colistin (polymyxin E), are a potent class of polypeptide antibiotics. Despite the clinical efficacy of colistin against Gram-negative bacteria, it is recommended for employment as an antibiotic of last resort, both to avoid resistance and, importantly, due to dose-limiting nephro- and neurotoxicity [7]. To reduce this toxicity and optimise antimicrobial activity, drug absorption and target specificity, several novel derivatives of polymyxin antibiotics are being developed [8,9,10,11]. Structural modifications have involved the N-terminal fatty acyl moiety or Dab side chains and demonstrated the importance of Dab at residue five in antimicrobial activity [12]. Progression to clinical trials of these polymyxin derivatives has, however, been limited due to their narrow spectrum of antimicrobial activity, and their cytotoxicity/poor tolerability in animal studies [13].

Polymer therapeutics have emerged as a promising strategy to combat antimicrobial resistance, particularly when used to reinstate “old” antibiotics [14]. Conjugation of an antibiotic to a water-soluble polymer offers many advantages compared to small molecule drugs, including reduced toxicity/immunogenicity, prolonged plasma half-life and improved pharmacodynamic targeting through the enhanced permeability and retention (EPR) effect [15]. Colistin has previously been conjugated to both, dextrin [16] and poly(ethylene glycol) (PEG) [17], however, complete restoration of antibiotic activity was not achieved after amylase-unmasking of dextrin–colistin conjugates, presumably due to the presence of oligosaccharides attached to the colistin amine groups [18]. The use of alternative conjugation chemistry may offer the opportunity to optimise reinstatement of antibiotic activity at sites of infection/inflammation [19]. Moreover, conjugation of the antibiotic to bioactive polysaccharides affords the opportunity to deliver anti-infective bi-functional polymer therapeutics.

Although alginates, like dextrin, are recognised as non-toxic by the Food and Drug Administration (FDA), their large molecular weight and lack of mammalian, alginate-degrading enzymes has limited their use in protein/peptide conjugation. More recently, a low molecular weight alginate oligosaccharide (OligoG, Mn 3200 g/mol), was extracted as a sodium salt from marine algae (*Laminaria hyperborea*) with >85% of residues being composed of α-L-guluronic acid. Although OligoG has no (MIC) value, it inhibits bacterial growth, adherence and biofilm development, and potentiates the activity of antibiotics against Gram-negative MDR pathogens [20,21,22,23,24]. This low molecular weight alginate also possesses hydroxyl and carboxyl functional groups that can be used for drug conjugation.

We hypothesised that conjugation of guluronic-rich, low molecular weight alginates to antibiotics, such as polymyxins, could create a bi-functional antibiotic polymer therapeutic [25]; combining the antimicrobial properties of both the antibiotic and the alginate, while simultaneously reducing systemic toxicity of the antibiotic, and facilitating size-dependent targeting by the EPR effect at the site of infection. Polymyxins were chosen as a model drug because previous studies have demonstrated that OligoG can enhance the antimicrobial efficacy of colistin against MDR, Gram-negative *P. aeruginosa* both in vitro and in vivo [26].

The aim of the study was to generate and characterise a bi-functional polymyxin conjugate using OligoG to optimise the antimicrobial function of these last resort antibiotics. A range of OligoG-polymyxin conjugates were synthesised and their physicochemical properties, in vitro cytotoxicity and biological activity characterised. Antimicrobial activity was assessed using MIC assays, growth curves, confocal laser scanning microscopy (CLSM) imaging and “time-to-kill” (TTK) studies.

## 2. Materials and Methods

### 2.1. Materials

OligoG CF-5/20 and the high molecular weight alginate PRONOVA UP MVG (>60% guluronic acid and Mw of 200,000 g/mol) were provided by AlgiPharma AS (Sandvika, Norway). The LIVE/DEAD^®^ Baclight^TM^ Bacterial Viability kit was from Invitrogen Molecular Probes (Paisley, UK). Pullulan gel filtration standards were from Polymer Laboratories (Church Stretton, UK). All chemicals were obtained from either Fisher Scientific (Loughborough, UK) or Sigma-Aldrich (Poole, UK) unless otherwise stated and were of analytical grade.

### 2.2. Cell Lines and Cell Culture

Human kidney proximal tubule cells (HK-2) were obtained from the American Type Culture Collection (ATCC) (Manassas, VA, USA) and screened to be free of mycoplasma contamination before use. Keratinocyte serum-free (K-SFM) medium (with l-glutamine), bovine pituitary extract (BPE, 0.05 mg/mL), human recombinant epidermal growth factor (EGF, 5 ng/mL), 0.05% *w*/*v* trypsin-0.53 mM ethylenediaminetetraacetic acid (EDTA) were from Invitrogen Life Technologies (Paisley, UK).

### 2.3. Bacterial Isolates and Growth Media

The bacterial strains (Table S1) used have been previously described [20,27]. Bacteria were grown on either tryptone soy agar (TSA) or blood agar (BA) plates supplemented with 5% *v*/*v* defibrinated horse blood. Bacterial overnight cultures were grown in tryptone soy (TS) broth and Mueller–Hinton (MH) broth was used for susceptibility testing. All media were from LabM (Bury, UK). Artificial sputum (AS) medium was prepared as previously described by Pritchard et al. [24].

### 2.4. Synthesis of OligoG–Polymyxin Conjugates

**Scheme 2:**
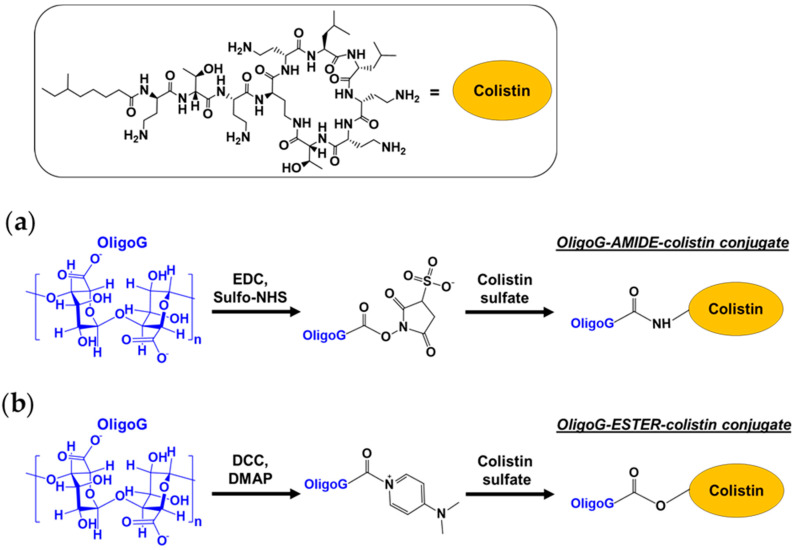
Schematic showing steps in the synthesis of the OligoG–colistin conjugate. (**a**) Using an amide linker (OligoG–A–colistin conjugate). (**b**) Using an ester linker (OligoG–E–colistin conjugate).

To synthesise amide (A)-linked conjugates (Scheme 2a), OligoG (1000 mg, 0.3 mmol), 1-ethyl-3-(3-dimethylaminopropyl) carbodiimide hydrochloride (EDC; 96.8 mg, 0.5 mmol) and N-hydroxysulfosuccinimide (sulfo-NHS; 109.6 mg, 0.5 mmol) were dissolved under stirring (15 min at 21 °C) in distilled water (dH_2_O; 10 mL). To this, colistin sulphate (146.7 mg, 0.1 mmol) or polymyxin B (144.4 mg, 0.1 mmol) was added followed by drop-wise addition of NaOH (0.5 M) until pH 8 was reached. The reaction mixture was stirred for 2 h at 21 °C, then stored at −20 °C prior to purification.

To synthesise ester (E)-linked conjugates (Scheme 2b), OligoG (1000 mg, 0.3 mmol), N,N′-dicyclohexyl carbodiimide (DCC; 64.5 mg, 0.3 mmol), 4-dimethylaminopyridine (DMAP; 6.4 mg, 0.05 mmol) and colistin sulphate (146.7 mg, 0.1 mmol) or polymyxin B (144.4 mg, 0.1 mmol) were dissolved while stirring overnight at 21 °C in anhydrous DMSO (10 mL). The reaction was stopped by pouring the mixture into excess chloroform (~100 mL). Formed precipitates were collected by filtration and dissolved in dH_2_O (10 mL), then stored at −20 °C prior to purification.

### 2.5. Purification of OligoG–Polymyxin Conjugates

OligoG–polymyxin conjugates were purified from the reaction mixture by fast protein liquid chromatography (FPLC) using an AKTA Purifier system (GE Healthcare; Amersham, UK) connected to a prepacked Superdex 75 16/600 GL column with a UV detector and a fraction collector (Frac-950). Data analysis was performed using Unicorn 5.31 software (2011; GE Healthcare; Amersham, UK). Samples (2 mL) were injected into a 2 mL loop using phosphate buffered saline (PBS) buffer (pH 7.4) as a mobile phase at 1 mL/min. Fractions were collected, pooled and lyophilised. Then, conjugates were re-dissolved in a minimal volume of dH_2_O and dialysed (1000 g/mol cut-off) against 5 × 1 L dH_2_O to remove PBS salts. The final conjugates were lyophilised and stored at −20 °C.

### 2.6. Characterisation of OligoG–Polymyxin Conjugates

Size exclusion chromatography with multi-angle light scattering detection (SEC-MALS) or refractive index detection (SEC-RI), were used to measure the approximate molecular weight and polydispersity of the conjugates. SEC-MALS was performed at ambient temperature on an HPLC system consisting of a solvent reservoir, on-line degasser, automatic sample injector, HPLC isocratic pump, pre-column and serially connected columns (TSKgel 4000 and 2500 PWXL). The column outlet was connected to a Dawn HELEOS-II multi-angle laser light scattering photometer (Wyatt, MO, USA) (λ0 = 663.8 nm) followed by a Shodex RI-501 RI detector. The eluent was 0.15 M NaNO3 with 0.01 M EDTA, pH 6.0 and the flow rate was 0.5 mL/min. Samples (10 mg/mL) were filtered (pore size 0.45 μm) before injection and analysed twice with injection volumes of 25 and 50 µL. A weighted specific refractive index increment (dn/dc) value was calculated from the % *w*/*w* colistin using dn/dc = 0.150 and 0.185 for alginate and colistin, respectively. Data were collected and processed using the Astra software (version 7.3.0; Wyatt, USA).

The SEC-RI system consisted of two TSK gel columns (G5000PWXL and G3000PWXL) (Tosoh, Germany) in series connected to a Gilson 133 differential refractometer (Middleton, WI, USA). Samples were prepared in PBS (3 mg/mL) and eluted using PBS (pH 7.4) as the mobile phase at a flow rate of 1 mL/min. Cirrus GPC software (version 3.2, 2006) from Polymer Laboratories (Church Stretton, UK,) was used for data analysis. Molecular weight was determined relative to pullulan molecular weight standards.

The FPLC system described above, connected to a Superdex 75 (10/300 GL) column, was also used to assess conjugate purity. Samples (3 mg/mL in PBS) were injected into a 100 µL loop at 0.5 mL/min. The area under the curve was used to estimate the percentage of free and conjugated antibiotic. The total polymyxin content of conjugates was determined by bicinchoninic acid (BCA) assay using colistin sulphate or polymyxin B standards.

Before and after OligoG conjugation, the number of available primary amine groups on colistin and polymyxin B was determined using the ninhydrin assay. First, a lithium acetate buffer (4 M) was prepared by dissolving lithium acetate dihydrate 40.81% *w*/*v* in dH_2_O. Acetic acid (glacial) was added to reach pH 5.2 before adjusting the final volume. Next, ninhydrin reagent was prepared by dissolving ninhydrin 2% *w*/*v* and hydrindantin 0.3% *w*/*v* in 7.5 mL of DMSO and 2.5 mL of lithium acetate buffer. Test compounds (86 µL) were diluted with ninhydrin reagent (1:1) and heated in a water bath (100 °C) for 15 min. Samples were subsequently cooled to room temperature and mixed with 50% *v*/*v* ethanol solution (130 µL). Then, aliquots (100 µL) were transferred into the wells of a 96-well microtitre plate and analysed spectrophotometrically at 570 nm. Calibration of the assay was achieved using ethanolamine (0–0.1158 mM).

NMR spectroscopy was used to confirm OligoG–polymyxin conjugation (Supplementary Materials).

### 2.7. Drug Release of OligoG–Polymyxin Conjugates

To compare the rate of degradation of OligoG–polymyxin conjugates, solutions were prepared (3 mg/mL) in either (i) PBS at pH 5, (ii) PBS at pH 7, or (iii) PBS at pH 7 containing alginate lyase from *Sphingobacterium multivorum* (1 U/mL) and incubated at 37 °C for 0, 2, 4, 6, 24 and 48 h. Upon collection, samples were immediately snap-frozen in liquid nitrogen and stored at −20 °C. Time-dependent changes in molecular weight and free polymyxin content were determined by SEC-RI and FPLC, respectively.

### 2.8. Characterisation of In Vitro Toxicity

A 3-(4,5-dimethylthiazol-2-yl)-2,5-diphenyltetrazolium bromide (MTT) assay was used to measure cell viability and proliferation of HK-2 cells. Cells were seeded into sterile 96-well microtitre plates at 1 × 10^5^ cells/mL (100 µL/well) and allowed to adhere for 24 h at 37 °C. The following day, the old medium was replaced with test compounds (0–1 mg/mL polymyxin base) dissolved in filter-sterilised K-SFM. After 67 h incubation at 37 °C, filter-sterilised MTT solution (20 µL of a 5 mg/mL solution in PBS) was added to each well and incubated for a further 5 h at 37 °C. Finally, the medium was carefully removed, and the formazan crystals were solubilised in DMSO (100 µL) for 30 min. Absorbance was measured at 550 nm using a Fluostar Omega microplate reader. The results are stated as percentage cell viability compared with the untreated control cells. Data are expressed as mean ± SEM (*n* = 18).

Release of the cytokine, tumour necrosis factor alpha (TNFα), by HK-2 cells (1 × 10^5^ cells/mL) after exposure to free- and OligoG-conjugated antibiotic (0–1 mg/mL polymyxin base) was assessed using an enzyme-linked immunosorbent assay (ELISA) kit. After 72 h incubation, the 96-well microtitre plates were centrifuged (226× *g*, 3 min), the supernatant was collected, diluted with reagent diluent (1:1) and analysed with the TNFα ELISA kit according to the manufacturer’s instructions (Fisher Scientific; Loughborough, UK). Plates were analysed spectrophotometrically at 450 nm. In parallel, 100 µL of K-SFM was added to the wells of the centrifuged plates containing cells and MTT assays were performed. A standard curve was used to calculate TNFα concentrations in the test samples, which were then multiplied by the dilution factor (×2) and divided by cell viability for each drug concentration (from the MTT assay). Two outliers were identified and removed using robust regression and the outlier removal (ROUT) method (Q coefficient = 0.2%). Data are expressed as mean ± SEM (*n* = 6).

### 2.9. Antimicrobial Activity of OligoG–Polymyxin Conjugates

The minimum inhibitory concentration (MIC) of colistin (as sulphate salt) and polymyxin B and their conjugates was determined using the broth microdilution method in MH broth in accordance with standard guidelines [28]. Test organisms were suspended in MH broth (100 µL, 5 × 10^5^ colony forming units (CFU)/mL) and incubated in 96-well microtitre plates in serial two-fold dilutions of the test compounds. The MIC was defined as the lowest concentration of test compound that produced no visible growth after 16–20 h. Results were expressed as mode (*n* = 3). For the purpose of calculating selectivity index (SI), MIC values lower than 0.008 were taken as the lowest concentration tested. Selective activities of the polymyxins and OligoG–polymyxin conjugates were calculated as follows:

Selectivity index (SI) = IC_50_ (μg/mL)/MIC (μg/mL).

To investigate whether alginate oligomer degradation is required for antimicrobial activity, MIC assays were also conducted in the presence of alginate lyase (1 and 10 U/mL), whereby alginate lyase was added to the MH broth during microtitre plate set up. In addition, alginate oligomer–colistin conjugates (3 mg/mL) were incubated in PBS at pH 7 containing bacterial alginate lyase (1 and 10 U/mL) at 37 °C for 24 h, before preparing microtitre plates as described above.

To more closely mimic in vivo environmental conditions, the antimicrobial activity of test compounds was studied in the presence of mucin, by supplementing MH broth with porcine stomach (type II) mucin (0.2 and 2% *w*/*v*) and used to set up the 96-well plates according to the standard MIC protocol. To account for turbidity caused by mucin, resazurin dye solution (30 µL, 0.01% *w*/*v* in dH_2_O) was added to each well and incubated for a further 3 h at 37 °C. Colour changes were observed and recorded.

To study the antimicrobial efficacy of test compounds under more clinically relevant conditions, the MIC protocol was performed using AS medium instead of MH broth. The plates were incubated with resazurin as described above.

A checkerboard assay was used to assess synergy of test compounds with azithromycin dihydrate. Here, stock solutions of test compounds (8 × MIC) and serial two-fold dilutions of azithromycin dihydrate (16–1/16 × MIC) were freshly prepared in MH broth. Test compound solutions (100 µL) were placed in the wells of row 1, then serially diluted along the ordinate with MH broth. Serially diluted azithromycin dihydrate solutions (50 µL) were then added to the wells in decreasing concentration along the abscissa. Each microtitre well was inoculated with the test organism (5 × 10^5^ CFU/mL) and incubated at 37 °C for 20 h. The fractional inhibitory concentration index (FICI) was calculated by comparing the MIC values of the individual agents with the MIC value of the combined treatments [29]. Drug combinations were considered synergistic when the mean FICI was ≤0.5, additive when the FICI was between 0.5 and 2, indifferent when the FICI was between 2 and 4, and antagonistic when the FICI was ≥4 [30]. Results were expressed as median values (*n* = 3).

To study the effect of test compounds on bacterial pharmacokinetic profiles, 96-well microtitre plates were set up according to the standard MIC protocol, then placed in a Fluostar Omega Microplate Reader at 37 °C, and absorbance at 600 nm was measured hourly for 48 h. Results were expressed as mean values (*n* = 3). Unconjugated colistin plus OligoG, OligoG and the high molecular weight, biologically inactive alginate, PRONOVA, at equivalent concentrations used in amide-linked or ester-linked conjugates, were used as controls.

### 2.10. Anti-Biofilm Activity of OligoG–Polymyxin Conjugates

To analyse the effect of test compounds on biofilm formation, solutions of test compounds in MH broth were inoculated (1:10) with *P. aeruginosa* R22 (standardised to 10^7^ CFU/mL) in a Greiner glass-bottomed optical 96-well plate. The plate was then wrapped in parafilm and incubated (37 °C, 20 rpm) for 24 h. The supernatant was carefully removed and replaced with 10% *v*/*v* LIVE/DEAD stain in PBS prior to imaging. CLSM of Syto 9 (λ_ex_/λ_em_ maximum, 480/500 nm) and propidium iodide (λ_ex_/λ_em_ maximum, 490/635 nm) was performed using a Leica SP5 confocal microscope with ×63 lens (under oil) and a step size of 0.79 µm. Z-stack CLSM images were analysed using COMSTAT image analysis software [31] and results were expressed as mean ± SEM (*n* = 15).

### 2.11. Pharmacokinetic–Pharmacodynamic (PK–PD) Model

A two-compartment static dialysis bag model (adapted from Azzopardi et al. [32]) was used to study the PK–PD profile of OligoG–colistin conjugates. First, the ability of the dialysis membrane to control diffusion of test compounds was assessed. The inner compartment (IC) contained OligoG–colistin or colistin sulphate (10 mg/mL colistin base; 5 mL) in PBS and the outer compartment (OC) contained sterile PBS (15 mL). The aseptically sealed beaker was incubated (37 °C, 70 rpm) for 48 h. Samples were collected at various time points from each compartment and stored at −20 °C prior to analysis of colistin content by BCA assay.

A modified experimental set-up was used to investigate the concentration- and time-dependent antimicrobial activity of test compounds using a TTK assay (48 h). Here, the total volume in the system was considered, with the IC containing test compounds at MIC (colistin sulphate 0.25 µg/mL; OligoG–A–colistin 0.125 µg/mL colistin base; OligoG–E–colistin 0.125 µg/mL colistin base) or 2 × MIC (OligoG–A–colistin 0.25 µg/mL colistin base; OligoG–E–colistin 0.25 µg/mL colistin base) in PBS. The OC contained MH broth inoculated with *A. baumannii* 7789 (5 × 10^5^ CFU/mL). Samples were collected from the OC (0, 2, 4, 6, 24 and 48 h) and colony counts (CFU/mL) determined using drop counts. Treatments were considered bactericidal if the reduction in viable bacterial counts was ≥3 log_10_ CFU/mL (equivalent to 99.9% of the initial inoculum) and bacteriostatic if the decrease was <3 log_10_ CFU/mL [33,34]. Growth (no test compounds) and sterility (no bacteria) controls were also performed.

### 2.12. Statistical Analysis

GraphPad Prism (version 6.01, 2012; San Diego, CA, USA) was used for statistical analysis. Statistical significance was indicated by *, where * *p* < 0.05, ** *p* < 0.01, *** *p* < 0.001 and **** *p* < 0.0001. Analysis of variance (ANOVA) was used to evaluate multiple group comparisons (*n* ≥ 3) followed by Dunnett’s post hoc test to account for multiple comparisons.

## 3. Results

### 3.1. Synthesis and Characterisation of OligoG–Polymyxin Conjugates

The characteristics of the OligoG–antibiotic conjugates synthesised in this study are summarised in Table 1 and Table S2. Polymyxin B conjugates typically showed less drug loading (6.1–8% *w*/*w*) than the colistin conjugates (8.1–12.9% *w*/*w*). SEC-MALS, SEC-RI and FPLC analysis confirmed the presence of high molecular weight conjugates with <6% unbound drug (Figures S1 and S2, Table S3). The mean molecular weight of amide- and ester-linked conjugates (measured by SEC-MALS) was 8200–12,800 g/mol and 5200–6200 g/mol, respectively. The ninhydrin assay indicated that 2–4 amine groups were used for binding to OligoG via amide conjugation. Diffusion-ordered spectroscopy (DOSY) NMR confirmed covalent coupling of OligoG to colistin. Signals corresponding to OligoG and colistin in the samples had the same diffusion coefficient (1.26 × 10^−10^ m^2^/s), indicative of covalent coupling (Figure S3). OligoG–A–colistin conjugate samples appeared to contain some free OligoG while the DOSY spectrum for OligoG–E–colistin conjugate showed the presence of both, free OligoG and colistin in the sample.

### 3.2. Stability of OligoG–Polymyxin Conjugates

Both ester- and amide-linked conjugates of OligoG–colistin and OligoG–polymyxin B incubated in PBS at either pH 5 or pH 7 showed no significant decrease in molecular weight (Figure S4). Conjugates were slightly less stable at pH 7, compared to pH 5. Conversely, alginate lyase effectively triggered ~30% of colistin and ~90% of polymyxin B release (increase in % free drug) within 24 h from these conjugates at 1 U/mL. There was little difference in total drug release between amide- and ester-linked conjugates.

### 3.3. Cytotoxicity of OligoG–Polymyxin Conjugates

**Figure 1:**
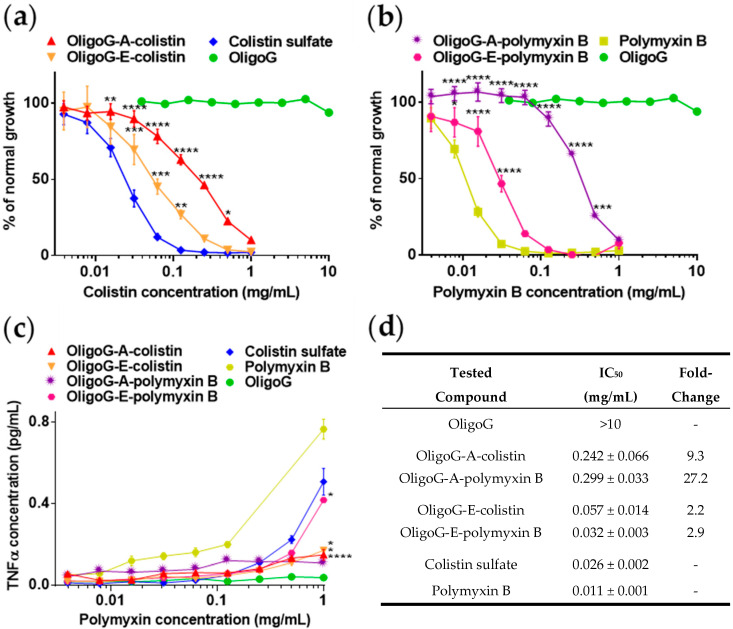
In vitro cytotoxicity of OligoG–polymyxin conjugates in human kidney proximal tubule cells (HK-2) cells. Cell viability determined by 3-(4,5-dimethylthiazol-2-yl)-2,5-diphenyltetrazolium bromide (MTT) assay after 72 h incubation. (**a**) Colistin sulphate. (**b**) Polymyxin B. Data are presented as mean % of untreated control ± SEM (*n* = 18). (**c**) Tumour necrosis factor alpha (TNFα) release in HK-2 cells after incubation with OligoG–polymyxin conjugates for 72 h (±SEM; *n* = 6). (**d**) Half maximal inhibitory concentration (IC_50_) values (±SEM) and fold-change (MTT assay) of tested compounds in HK-2 cells. Significance is indicated by *, where * *p* < 0.05, ** *p* < 0.01, *** *p* < 0.001, **** *p* < 0.0001 compared to colistin sulphate or polymyxin B controls. Abbreviations: A, amide; E, ester.

The concentration-dependent cytotoxicity of unmodified antibiotics, OligoG and OligoG-polymyxin conjugates in HK-2 cells is shown in Figure 1. OligoG was not cytotoxic at <10 mg/mL. Cytotoxicity was greatest for the free drugs (colistin sulphate half maximal inhibitory concentration (IC_50_ = 0.026 mg/mL), polymyxin B (0.011 mg/mL)); slightly reduced by ester conjugation (OligoG–E–colistin (IC_50_ = 0.057 mg/mL), OligoG–E–polymyxin B (0.032 mg/mL)); and significantly reduced for the amide-linked conjugates (OligoG–A–colistin (IC_50_ = 0.242 mg/mL), OligoG–A–polymyxin B (0.299 mg/mL)). ELISA showed that the unmodified antibiotics induced greater TNFα release than the conjugates (Figure 1c). OligoG–E–polymyxin B caused the highest release of TNFα, compared to the other conjugates, but this was still lower than the unmodified drug.

### 3.4. Antimicrobial Activity of OligoG–Polymyxin Conjugates

The effects of conjugation on antimicrobial efficacy against a range of Gram-negative pathogens varied between the conjugates and antibiotic (Table 2). Whilst ester-conjugation resulted in similar (≤2-fold differences) or decreased MIC values for OligoG–colistin and –polymyxin B conjugates, the amide-linked conjugates demonstrated increased MIC values. This effect was particularly evident for the polymyxin B conjugates, where MICs were increased by 4- to 32-fold. OligoG conjugation did not improve the bactericidal efficacy of colistin in colistin-resistant strains (Table 2). Both OligoG–polymyxin conjugates exhibited substantially improved selectivity for Gram-negative bacteria in comparison to mammalian cells, compared to unmodified colistin sulphate (1.7–4.7-fold) and polymyxin B (2.3–4.1-fold) (Table S4).

The antimicrobial activity of the conjugates was also assessed in the presence of alginate lyase or following pre-incubation with alginate lyase (Table S5), where no significant change was observed for either amide- or ester-bonded conjugates.

In contrast, when mucin was added to the broth, antimicrobial activity of OligoG–polymyxin conjugates decreased in a dose-dependent manner (Table S6). The presence of unconjugated OligoG with colistin sulphate or polymyxin B did not alter the antimicrobial activity of the free antibiotic.

Differences in antimicrobial activity of both, free- and OligoG-bound antibiotics were observed in AS medium compared to MH broth (Table S7). In most cases, using the checkerboard assay, an indifferent or additive effect was observed when azithromycin dihydrate was combined with OligoG–colistin or colistin sulphate (Table S8). Generally, OligoG conjugation did not alter the efficacy of the antibiotic combination, except for *A. baumannii* 7789, where the additive effect of colistin sulphate + azithromycin dihydrate (FICI = 1.14) became indifferent when azithromycin dihydrate was combined with OligoG–colistin conjugates (FICI >2.5). However, the combination of azithromycin dihydrate with OligoG–E–colistin resulted in a synergistic effect for the *E. coli* National Collection of Type Culture (NCTC) 10418 isolate (FICI = 0.46).

**Figure 2:**
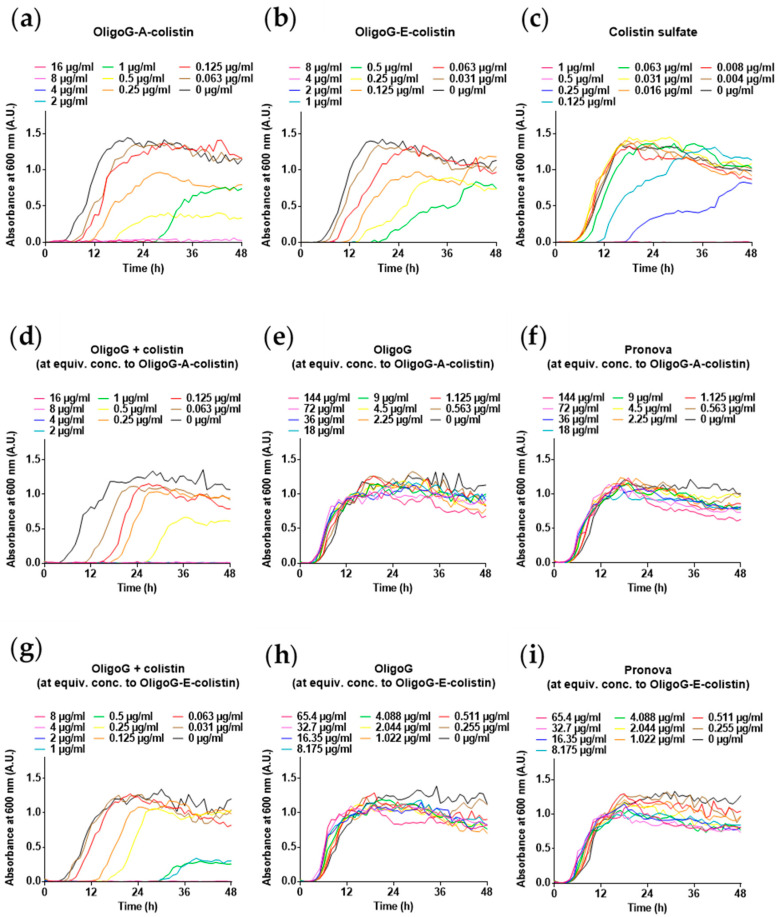
Bacterial growth curves for *P. aeruginosa* MDR 301 (48 h) in the presence of the following antimicrobials. (**a**) OligoG–A–colistin conjugate. (**b**) OligoG–E–colistin conjugate. (**c**) Colistin sulphate. (**d**) Colistin sulphate plus OligoG control for amide-linked conjugate. (**e**) OligoG control for amide-linked conjugate. (**f**) PRONOVA control for amide-linked conjugate. (**g**) Colistin sulphate plus OligoG control for ester-linked conjugate. (**h**) OligoG control for ester-linked conjugate. (**i**) PRONOVA control for ester-linked conjugate. Controls were used at the equivalent concentrations used in the corresponding amide- or ester-linked conjugates (*n* = 3). Abbreviations: A, amide; E, ester.

Bacterial growth curves of *P. aeruginosa* MDR 301 (Figure 2) showed that the OligoG–colistin conjugates delayed bacterial growth in a concentration-dependent manner (indicated by the longer lag-phase), and exponential growth was similarly slower compared to the untreated control. Growth inhibition (up to >48 h) was noted for the OligoG–colistin conjugates at ≥2 × MIC, while higher equivalent concentrations of colistin sulphate were required (≥8 × MIC) to achieve comparable efficacy. Neither amide- nor ester-linked conjugates had an effect on time to onset of bacterial growth. Both, OligoG–A–colistin and OligoG–E–colistin conjugates, at their MIC (1 and 0.5 µg/mL colistin base, respectively), demonstrated a delayed lag phase of >24 h and >18 h, respectively. Typically, colistin covalently conjugated to OligoG showed similar activity to the combined mixture of unconjugated colistin and OligoG at equivalent concentrations. Furthermore, neither OligoG nor Pronova alone had any significant effect in reducing bacterial growth, when an equivalent concentration to that contained in OligoG–colistin conjugates was used.

### 3.5. Anti-Biofilm Activity of OligoG–Polymyxin Conjugates

**Figure 3:**
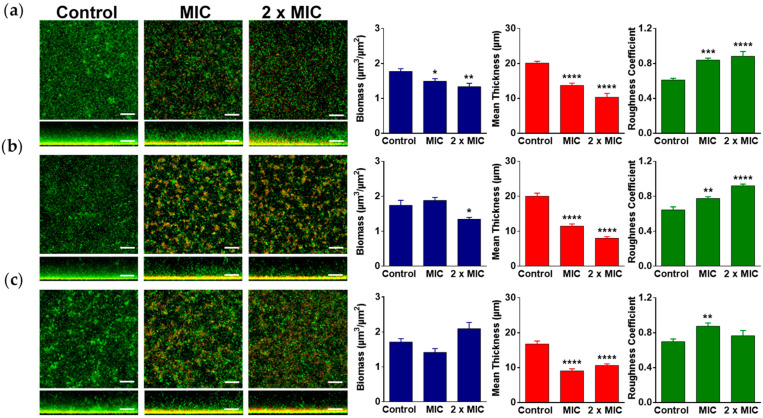
Biofilm formation assay showing LIVE/DEAD (green/red, respectively) stained confocal laser scanning microscopy (CLSM) images (aerial and cross-sectional views, scale bar, 35 μm) of *P. aeruginosa* R22 biofilms grown for 24 h in the presence of (**a**) OligoG–A–colistin (MIC, 2 μg/mL colistin base), (**b**) OligoG–E–colistin (MIC, 1 μg/mL colistin base) and (**c**) colistin sulphate (MIC, 0.5 μg/mL colistin base). Corresponding COMSTAT analysis of the CLSM images is also shown (±SEM; *n* = 15). Significance is indicated by *, where * *p* < 0.05, ** *p* < 0.01, *** *p* < 0.001, **** *p* < 0.0001 compared to an untreated control.

CLSM of biofilms grown in the presence of free and OligoG-bound colistin (≥MIC) showed a marked effect on biofilm formation (Figure 3). For example, the OligoG–E–colistin conjugate, at its MIC (1 µg/mL colistin base), caused obvious bacterial clumping, disruption of biofilm structure, and cell death (as noted by increased numbers of red cells). COMSTAT analysis revealed a significant reduction in biofilm thickness for all treatments (≥MIC) (*p* < 0.05) and biofilm roughness was significantly increased by OligoG–colistin conjugates (≥MIC) and colistin sulphate (MIC) treatments (*p* < 0.05). Both OligoG–colistin conjugates (2 × MIC) significantly reduced biofilm biomass compared to untreated control (*p* < 0.05), whereas no significant change was observed with colistin sulphate (up to 2 × MIC).

### 3.6. Pharmacokinetic–Pharmacodynamic (PK–PD) Model

**Figure 4:**
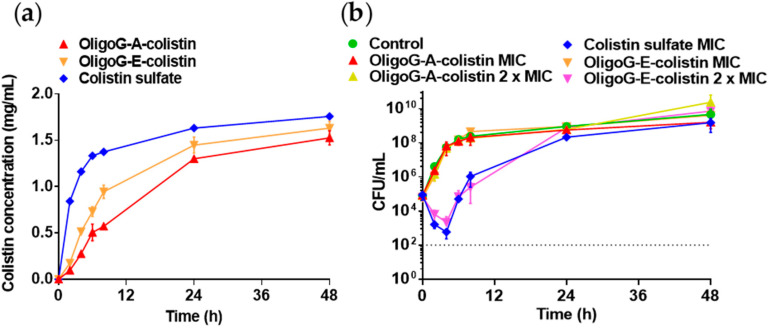
Pharmacokinetic–pharmacodynamic (PK–PD) model to compare diffusion of free- and OligoG-conjugated colistin. (**a**) Change in colistin concentration in the outer compartment over 48 h (measured by BCA assay). Data are expressed as mean ± SD (*n* = 3). (**b**) Viability count of *A. baumannii* 7789 in a “time-to-kill” (TTK) model. Data represent mean colony forming units (CFU) ± SD (*n* = 3). Dotted line, lower limit of detection (10^2^ CFU/mL). Abbreviations: A, amide; E, ester.

In the PK-PD model (Figure 4), when antibiotic was placed in the IC, colistin diffused more rapidly than the OligoG–colistin conjugates (1 mg/mL in the OC was reached at 2.83 h (colistin) < 10.47 h (OligoG–E–colistin) < 17.35 h (OligoG–A–colistin)). Colistin sulphate, at MIC (0.25 µg/mL) and the OligoG–E–colistin conjugate at 2 × MIC (0.25 µg/mL colistin base) caused substantial bacterial killing after 4 h (<3 log_10_ CFU/mL) (Figure 4b). Both treatments caused a reduction in viable bacterial counts compared to the control (~5-fold lower) and the initial starting bacterial concentration (~2-fold lower). However, no antimicrobial effect was observed with the OligoG–A–colistin conjugate up to 2 × MIC.

## 4. Discussion

### 4.1. Rationale for Development of OligoG–Polymyxin Conjugates

In recent years, interest in developing polymyxin derivatives to improve the therapeutic index or provide activity towards bacterial strains that are not currently susceptible to polymyxins has grown considerably [35]. Structural modifications, such as removal or substitution of the N-terminal acyl chain, reduction in the number of positive charges, polymer conjugation and the introduction of hydrophobic residues, have all been explored in an attempt to improve activity, reduce adverse side effects and elucidate structure–activity relationships [36,37]. These studies have demonstrated the critical importance of the amphipathicity of polymyxin molecules for their antimicrobial activity, which stems from the charged Dab residues and hydrophobic tail. This study compared the activity and toxicity of OligoG–polymyxin conjugates containing reversible or irreversible linking chemistries, to improve the therapeutic index of the polymyxins.

To form an amide bond between OligoG and polymyxin antibiotics, the carboxyl groups of the polymer were first activated by EDC in the presence of sulfo-NHS to create a stable amine-reactive intermediate [38]. The resultant amine-reactive sulfo-NHS ester was then bound to primary amines on the polymyxins. We hypothesised that amide-linked conjugates would rely on degradation of the OligoG backbone by alginate lyase. There are no known mammalian enzymes capable of degrading alginate, but bacterial alginate lyase from *K. pneumoniae* and *P. aeruginosa* has been found in cystic fibrosis patients’ lungs [39] and could provide an opportunity for site-specific release of a therapeutic payload from an alginate conjugated drug. Although alginate lyases from these bacteria are generally considered to be mannuronate-specific, it has been shown that they may also demonstrate moderate to low activity towards guluronate [40]. Non-biodegradable polymers whose molecular weight is below the renal threshold (<40,000 g/mol) are expected to be readily excreted from the body [41], so the conjugates synthesised in the present study should be readily excreted by the kidney. Furthermore, sugar residues and/or linker groups that remain attached to the antibiotic Dab residues after polymer degradation, may result in reduced antimicrobial activity, as observed with amide-linked dextrin–colistin conjugates [18]. In parallel, ester-linked OligoG–polymyxin conjugates were formed using Steglich esterification. Here, the polymer carboxyl groups were activated by DCC, with DMAP as a catalyst [42]. The resultant *O*-acylisourea intermediate was then bound to hydroxyl groups on the polymyxins. Addition of DMAP as a catalyst compensates for hydroxyls being poorer nucleophiles than amines (which can cause spontaneous rearrangement of the *O*-acylisourea intermediate into undesirable *N*-acylurea) by reacting with *O*-acylisourea to form an acyl pyridinium species. Reaction of DMAP with *O*-acylisourea forms an acyl pyridinium intermediate that is unable to form intramolecular by-products but can react with a hydroxyl group to form an ester bond [43]. Ester-linkage permits complete release of the native antibiotic at low or high pH, and by enzymatic activity and reactive oxygen species at sites of infection [44]. Moreover, release of intact OligoG at the target site would restore intrinsic antimicrobial activity of the polymer, which would further enhance its antibiotic efficacy in vivo.

### 4.2. Physicochemical Characterisation of OligoG–Polymyxin Conjugates

Covalent attachment of OligoG to colistin was confirmed using several methods. DOSY is an indirect method that can detect if the polymer and peptide are chemically linked as they will have the same diffusion coefficient if bound together in solution [45] while SEC detects an increase in size caused by an increase in molecular weight. In a control experiment using SEC-MALS, OligoG with 10% *w*/*w* added colistin yielded almost identical RI chromatograms as pure OligoG, confirming that the observed shift in elution profile for the OligoG–colistin conjugates was not simply due to electrostatic interactions between OligoG and free colistin. DOSY confirmed successful conjugation using both amide and ester linkers. Nevertheless, DOSY also detected unbound colistin in the OligoG–E–colistin sample and SEC-MALS analysis did not show an increased molecular weight compared to free OligoG. This may be caused by hydrolysis of the ester linkage during measurement (in D_2_O at 25 °C), rather than insufficient removal of unreacted material, highlighting the importance of using multiple analytical methods to characterise these complex molecules.

### 4.3. Biological Characterisation of OligoG–Polymyxin Conjugates

Polymyxin antibiotics have been reported to cause severe nephrotoxicity in up to 53.5% of patients [46] due to extensive reabsorption of the drug by renal tubular cells [47]. OligoG–polymyxin conjugates in the present study exhibited a marked decrease in in vitro cytotoxicity in kidney cells when compared to unmodified antibiotics. As expected, ester-linked polymyxin conjugates were considerably more cytotoxic towards HK-2 cells compared to amide-linked conjugates. Since the positively charged Dab residues are known to mediate polymyxin toxicity, and 2–4 of these primary amines were used for irreversible (amide-linked) conjugation with OligoG, this result was unsurprising.

Antimicrobial efficacy of all OligoG–polymyxin conjugates was comparable to that of the parent antibiotic. However, attachment of OligoG in the bi-functional molecule was unable to overcome colistin resistance. For colistin-sensitive strains, conjugate MIC values were below the Clinical and Laboratory Standards Institute [48] and European Committee on Antimicrobial Susceptibility Testing [49] susceptibility breakpoints (≤2 µg/mL) for polymyxins. Importantly, ester-linked conjugates showed full retention of the antimicrobial activity of the free drug, while the antimicrobial activity of the amide-linked conjugates was reduced by more than two-fold, presumably because of residual sugars and/or linker groups on the antibiotic amine groups. Although these studies did not test the stability of the conjugates in bacterial growth medium, it is likely that hydrolysis of the ester bond would occur during the MIC assay incubation. This may explain the smaller decrease in antimicrobial activity seen with ester-linked conjugates compared to amide-linked ones. Greater selectivity of OligoG–A–polymyxin conjugates for Gram-negative bacteria in comparison to mammalian cells suggests better tolerability and reduced side effects in vivo and substantially improved efficacy at clinically relevant concentrations compared to the free antibiotic. In addition, studies with alginate lyase suggested that either OligoG degradation is not necessary for antibiotic activity, or that alginate oligomers are broken down by bacterial enzymes. Nevertheless, compared to dextrin–colistin conjugates described in previous studies [16], alginate oligomer–conjugates were significantly more potent (more than five-fold change). This may be due to the larger molecular weight of dextrin causing steric hindrance or the charge difference between the two polymers, but more likely, it can be attributed to the inherent biological activity of OligoG itself [20].

Sustained antibiotic release was demonstrated by slower bacterial growth in the presence of OligoG–colistin conjugates, which was dose-dependent. In this study, lower concentrations of OligoG–colistin conjugates, compared to free colistin, were required to inhibit bacterial growth which was sustained for up to 48 h. OligoG–E–colistin delayed the onset of bacterial growth for much longer than amide-linked conjugates, suggesting that, after systemic administration, OligoG–E–colistin conjugates might achieve better therapeutic activity in vivo. Similarly, when PEG was attached to colistin via a labile ester bond, sustained drug release led to equivalent or better antimicrobial activity against *A. baumannii* and *P. aeruginosa* isolates [17].

In a clinical setting, binding of colistin to sputum biomolecules (e.g., mucin) in the airways could negatively impact antibiotic effectiveness and availability. Indeed, Huang et al. [50] demonstrated >100-fold increase in MIC values of both, colistin and polymyxin B, when mucin was added to the bacterial culture medium. A four-fold increase in the MIC of colistin was also reported when the assay was conducted in AS medium instead of MH broth, thought to be caused by bacterial growth disruption, structural modifications of lipopolysaccharides or direct colistin–mucin interactions [24]. Although recent studies have demonstrated the ability of OligoG to bind mucin [51], conjugation of OligoG to colistin and polymyxin B did not affect the ability of the antibiotic to bind mucin or alter the effect of nutrient-deficient medium.

In practice, patients with severe infections of MDR pathogens are usually treated with combinations of two or more antibiotics to overcome or prevent drug resistance. When we combined OligoG–colistin conjugates with azithromycin dihydrate, an antibiotic that has previously shown enhanced efficacy in combination with OligoG [20], antimicrobial activity of the drug was enhanced, but only additively. Similarly, He et al. [52] reported additive effects in *P. aeruginosa* when they combined a low molecular weight alginate oligosaccharide (Mw < 10 kDa) with azithromycin, suggesting that the alginate oligosaccharide component of OligoG–colistin conjugates may be responsible for the additive effects observed in our study.

Chronic airway infections by *P. aeruginosa* affect more than 80% of CF patients and contribute to a progressive decline in lung function [53]. Marked disruption of *P. aeruginosa* biofilm formation was observed when they were grown in the presence of both OligoG–colistin conjugates, although only the ester-linked conjugate induced bacterial clumping (≥MIC) which might be associated with the higher cationic charge of colistin. This is in keeping with the findings of Powell et al. [23] who showed that OligoG, at concentrations ≥0.5%, caused *P. aeruginosa* aggregation, while higher concentrations (≥2%) caused significant disruption of bacterial biofilm formation and growth.

### 4.4. PK–PD Modelling

Drug TTK profiles and colistin release rate from amide- or ester-linked OligoG–colistin conjugates were investigated using an in vitro two-compartment PK–PD model. Predictably, diffusion of colistin, which was mirrored by a time-dependent increase in drug concentration in the OC, was substantially faster than the OligoG conjugates. When the ester-linked OligoG–colistin conjugate was contained in the IC, diffusion of colistin was more pronounced than when the amide-linked conjugate was tested, presumably due to the unstable nature of the ester bond. *A. baumannii* is an opportunistic pathogen that can causes serious infections often associated with multidrug resistant strains and has an 8.4–36.5% mortality rate [54]. In the present TTK study, although colistin sulphate at MIC (0.25 µg/mL colistin base) and the OligoG–E–colistin conjugate at 2 × MIC (0.25 µg/mL colistin base) exhibited rapid initial antimicrobial efficacy, marked bacterial re-growth was observed at 24 h. Previous studies have reported the impact of hetero-resistance of *A. baumannii* clinical isolates to colistin that allowed significant bacterial re-growth at 24 h at 32 × MIC [55] and 64 × MIC [56]. The reduction of viable bacterial counts by <3 log_10_ CFU/mL compared to the initial inoculum was indicative of bacteriostatic activity only. Similarly, bacteriostatic activity of colistin, at its MIC, towards *A. baumannii* clinical isolates has been demonstrated, indicating a 2-fold decrease in CFU/mL at 4–6 h post-dose [57]. Importantly, previous studies saw significant bactericidal activity of colistin when carbapenem-resistant *A. baumannii* isolates were treated with higher concentrations of the antibiotic (≥4 × MIC) [58]. Observations in the present study support the clinical limitations of conventional colistin therapy, due to concentration-dependent nephrotoxicity, which may limit the optimal dosing and efficacy of the antibiotic.

The findings of this study indicate that the ester-linked OligoG–colistin conjugate could be a suitable alternative to conventional colistin, as it demonstrated equivalent antimicrobial effectiveness (0.25 µg/mL colistin base) but exhibited significantly lower cytotoxicity in human kidney cells. Following systemic administration of colistimethate sodium (Colomycin^®^; prodrug of colistin), the plasma colistin concentration at steady-state is 0.5–4 µg/mL [59]. Clinically, nephrotoxicity is an important limiting factor to colistin dosing, therefore, a plasma concentration of 2 µg/mL is desirable to target bacterial pathogens with MIC values ≤1 μg/mL [60]. To avoid acute kidney injury, a maximum plasma concentration of 2.42 µg/mL is recommended [61]. Yet, the clinical susceptibility breakpoint for colistin against *Acinetobacter* spp. is 2 µg/mL [48,49], which gives it a narrow therapeutic index in vivo. Due to the EPR effect, OligoG–E–colistin conjugates are expected to accumulate within infected tissues, so higher antibiotic concentrations (>0.25 µg/mL colistin base, equivalent to >2 × MIC) could theoretically be achieved much quicker than with the unmodified antibiotic. Sustained release over 48 h and concentration-dependent antibacterial efficacy of colistin has been achieved using dextrin–colistin conjugates [32]. In that study, colistin was covalently linked to dextrin through an amide bond, so “unmasking” of antibiotic relied on α-amylase-mediated degradation of the polymer. The fact that the OligoG–A–colistin conjugate did not show any antimicrobial effect in the PK–PD model in this study could be attributed to the absence of alginate lyase in the culture medium or the presence of residual saccharides attached to colistin which would not be present on antibiotic released from the ester-linked conjugates. Recently, it has been demonstrated that, even after complete amylase degradation of dextrin in amide-linked dextrin-colistin conjugates, the colistin molecule was still attached to at least one linker with varying lengths of glucose units [18]. These findings suggest that complete “unmasking” or release of colistin is a pre-requisite for reinstatement of antibiotic activity.

Importantly, the therapeutic benefits of OligoG–colistin conjugates might have been underestimated by the in vitro assays. Passive accumulation of conjugates at sites of in vivo bacterial infection due to the EPR effect, alongside the local reduced pH, reactive oxygen species and esterase activity as well as alginate lyase could all promote the controlled release of the drug from the polymer, and might further enhance the efficacy of the drug in vivo and thus, reduce the doses required to eradicate infection.

## 5. Conclusions

This study has established, for the first time, the potential therapeutic benefits of using OligoG conjugation to reduce antibiotic toxicity, while maintaining antimicrobial activity against MDR Gram-negative bacterial pathogens. These studies also demonstrate that complete detachment of the polymer from the bioactive compound is required to restore its full biological efficacy, with residual sugars shown to impede complete regeneration of activity. As OligoG has been shown to enhance the antimicrobial activity of macrolides, tetracyclines and β-lactams antibiotics, against a range of MDR Gram-negative bacteria [20], OligoG conjugation might also improve the pharmacokinetics of other toxic, water-insoluble or otherwise undeliverable drugs. Polymer conjugates like the OligoG–polymyxins offer a novel approach to repurpose “old” antibiotics into safer, less toxic bi-functional compounds to meet the increasingly urgent need for new antimicrobial therapies.

## Figures and Tables

**Table 1:** Summary of the properties of the OligoG-polymyxin conjugates synthesised in this study.

Tested	Mw (g/mol) (PDI) by SEC-MALS	Polymyxin Content (% *w*/*w*)	Molar Ratio (per Colistin)	Conjugated NH_2_ per Molecule	Free Polymyxin (%)
OligoG–A–colistin					
Mean	9220 (1.3)	9.4	4.3	3.4	3.2
Range	8200–12,300 (1.2–1.4)	8.1–12.5	3.1–5.0	2.7–4.6	1.5–5.7
OligoG–E–colistin					
Mean	5550 (1.3)	10.9	3.8	N/A	2.7
Range	5200–5900 (1.2–1.3)	8.3–12.9	3.0–4.9	N/A	2.0–3.5
OligoG–A–polymyxin B					
Mean	10,950 (1.4)	7.1	6.0	2.0	1.6
Range	9100–12,800 (1.3–1.5)	6.1–8.0	5.1–6.8	1.9–2.0	1.6
OligoG–E–polymyxin B	6200 (1.2)	7.0	5.9	N/A	2.7

Abbreviations: A, amide; E, ester; PDI, polydispersity index (given in brackets); SEC-MALS, size exclusion chromatography with multi-angle light scattering detection; N/A, not applicable.

**Table 2:** Minimum inhibitory concentration (MIC) determinations of OligoG–polymyxin conjugates against a range of Gram-negative bacterial pathogens.

Isolate	Tested Compound MIC (μg/mL Drug Base)					
	Colistin Sulphate	Polymyxin B	OligoG–E–Colistin	OligoG–E–Polymyxin B	OligoG–A–Colistin	OligoG–A–Polymyxin B
*P. aeruginosa* R22	0.5	0.25	1	0.25	2	4
*P. aeruginosa* MDR 301	0.5	0.5	0.5	0.5	1	2
*P. aeruginosa* NH57388A	0.25	0.25	0.25	0.25	0.5	1
*P. aeruginosa* NCTC 10662	0.125	0.063	0.25	0.25	1	4
*K. pneumoniae* KP05 506	0.125	0.125	0.125	0.25	0.125	0.5
*K. pneumoniae* IR25	0.063	0.125	0.125	0.125	1	4
*A. baumannii* MDR ACB	0.5	0.125	**0.25**	**0.063**	1	2
*A. baumannii* 7789	0.25	0.125	**0.125**	0.5	**0.125**	0.5
*E. coli* AIM-1	<0.008	<0.004	0.008	0.016	0.008	0.063
*E. coli* IR57	0.125	0.5	0.25	0.5	0.125	2
*E. coli* 5702	0.031	0.063	0.031	0.063	0.063	0.25
*E. coli* NCTC 10418	0.125	0.25	0.5	0.25	0.25	4
*E. coli* PN21	8	8	16	8	32	32
*E. coli* PN25	8	4	8	4	32	32
*E. coli* PN26	0.125	0.125	0.25	0.25	0.5	0.5
*E. coli* ATCC 25922	0.25	0.5	1	0.5	1	16

Increased antimicrobial activity of conjugated polymyxin (MIC ≥ 1-fold lower compared to colistin sulphate or polymyxin B controls) is shown in bold. Abbreviations: A, amide; E, ester.

## Supplementary Materials

The following are available online at https://www.mdpi.com/1999-4923/12/11/1080/s1, Figure S1: Size exclusion chromatography with multi-angle light scattering detection (SEC-MALS) analysis of OligoG-conjugates (three different batches of OligoG–A–colistin (OAC) and one batch of OligoG–E–colistin (OEC)), showing overlaid refractive index chromatograms and corresponding Mw-time calibration lines. The injected mass was 250 µg for all samples. Abbreviations: A, amide; E, ester, Figure S2: SEC-MALS analysis of OligoG in the absence and presence of 10% *w*/*w* colistin, showing that they do not form strong complexes since the elution profile is identical, Figure S3: Diffusion-ordered spectroscopy (DOSY) of (a–c) three different batches of OligoG–A–colistin conjugates and (d) one batch of OligoG–E–colistin conjugate. The assignment of the unique signals for OligoG and colistin is indicated at the top of each panel and the red lines indicate the average diffusion coefficients of the molecules, Figure S4: Drug release of OligoG–polymyxin conjugates in phosphate buffered saline (PBS) at pH 5, pH 7 or pH 7 containing alginate lyase (AlgL). (a) Content of free polymyxin and (b) change in molecular weight were determined by fast protein liquid chromatography (FPLC) and size exclusion chromatography with refractive index detection (SEC-RI), respectively, over 48 h incubation. Abbreviations: A, amide; E, ester, Table S1: Gram-negative bacterial isolates used for characterisation of OligoG–polymyxin conjugates, Table S2: Physicochemical characteristics and batch details of OligoG–polymyxin conjugates used in this study, Table S3: Weight and number average molecular weights of OligoG and OligoG–colistin conjugates, Table S4: Selectivity index (SI) values of OligoG–polymyxin conjugates against a range of Gram-negative bacterial pathogens, Table S5: Microbiological efficacy (MICs) of OligoG–colistin conjugates in the presence of alginate lyase in Mueller–Hinton (MH) broth or after pre-incubation with alginate lyase against Gram-negative bacterial pathogens, Table S6: Microbiological efficacy (MICs) of polymyxins and antibiotic conjugates in the absence and presence of mucin against Gram-negative bacterial pathogens, Table S7: Comparison of the effect of growth medium (AS medium and MH broth) on antimicrobial activity (MIC determinations) of polymyxins and antibiotic conjugates, Table S8: Fractional inhibitory concentration index (FICI) values of OligoG–colistin conjugates or colistin in combination with azithromycin dihydrate, Methods: NMR spectroscopy.
