# Supplementary material for: Bi-Functional Alginate Oligosaccharide–Polymyxin Conjugates for Improved Treatment of Multidrug-Resistant Gram-Negative Bacterial Infections
Source: Pharmaceutics. 2020 Nov 11;12(11):1080. doi: 10.3390/pharmaceutics12111080 (PMC7696216; doi:10.3390/pharmaceutics12111080)
Supplement: Supplementary file 1 [file pharmaceutics-12-01080-s001.pdf]

pharmaceutics

Supplementary Materials: Bi-Functional Alginate Oligosaccharide–Polymyxin Conjugates for Improved Treatment of Multidrug-Resistant Gram-Negative Bacterial Infections

Joana Stokniene, Lydia C. Powell, Olav A. Aarstad, Finn L. Aachmann, Philip D. Rye, Katja E. Hill, David W. Thomas and Elaine L. Ferguson

1. Methods

NMR Spectroscopy

Samples (10 mg) were dissolved in 99.9 atom % D_2O (600 μL) and analysed by NMR at 25 °C on a Bruker AVIII-HD 800 spectrometer equipped with CP-TCI 5 mm cryoprobe (Bruker BioSpin AG; Fällanden, Switzerland). Diffusion-ordered spectroscopy (DOSY) was used to measure the diffusion of the OligoG conjugate products. A 2D DOSY was measured using a Bruker BioSpin stimulated echo pulse sequence with bipolar gradients (STEBPGP). Gradient pulses of 2 ms duration (δ) and 32 different strengths varying linearly from 0.03 to 0.57 Tm^{-1} were applied and the diffusion delay (Δ) was set to 80 ms. The spectra were recorded using the TopSpin software (version 3.5pl7) and analysed with the TopSpin software (version 4.0.7) (Bruker BioSpin AG; Fällanden, Switzerland).

2. Results

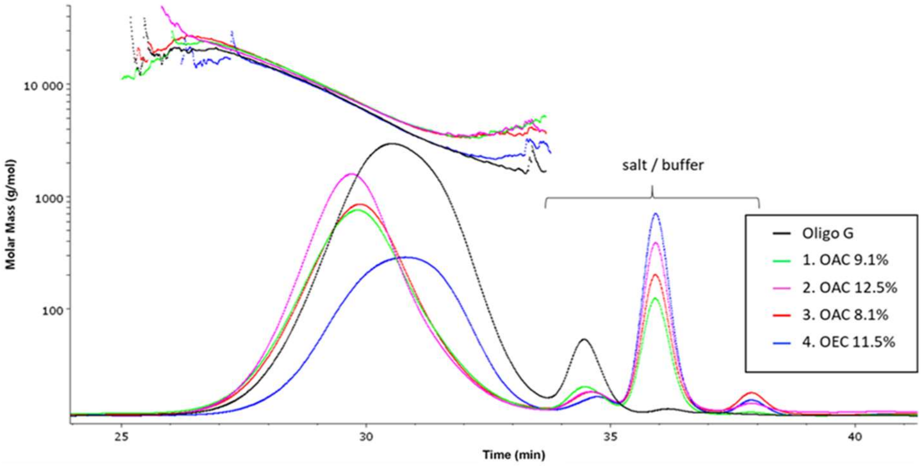

Figure S1. Size exclusion chromatography with multi-angle light scattering detection (SEC-MALS) analysis of OligoG-conjugates (three different batches of OligoG-A-colistin (OAC) and one batch of OligoG-E-colistin (OEC)), showing overlaid refractive index chromatograms and corresponding Mw-time calibration lines. The injected mass was 250 μg for all samples. Abbreviations: A, amide; E, ester.

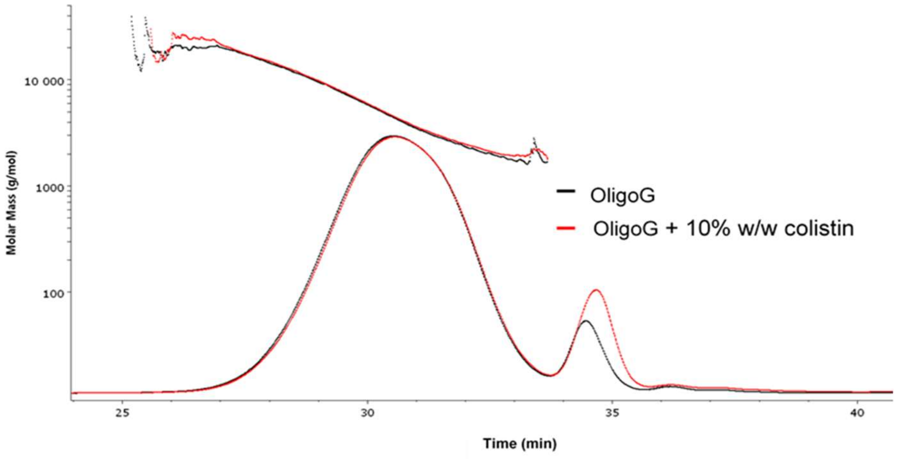

Figure S2. SEC-MALS analysis of OligoG in the absence and presence of 10% *w/w* colistin, showing that they do not form strong complexes since the elution profile is identical.

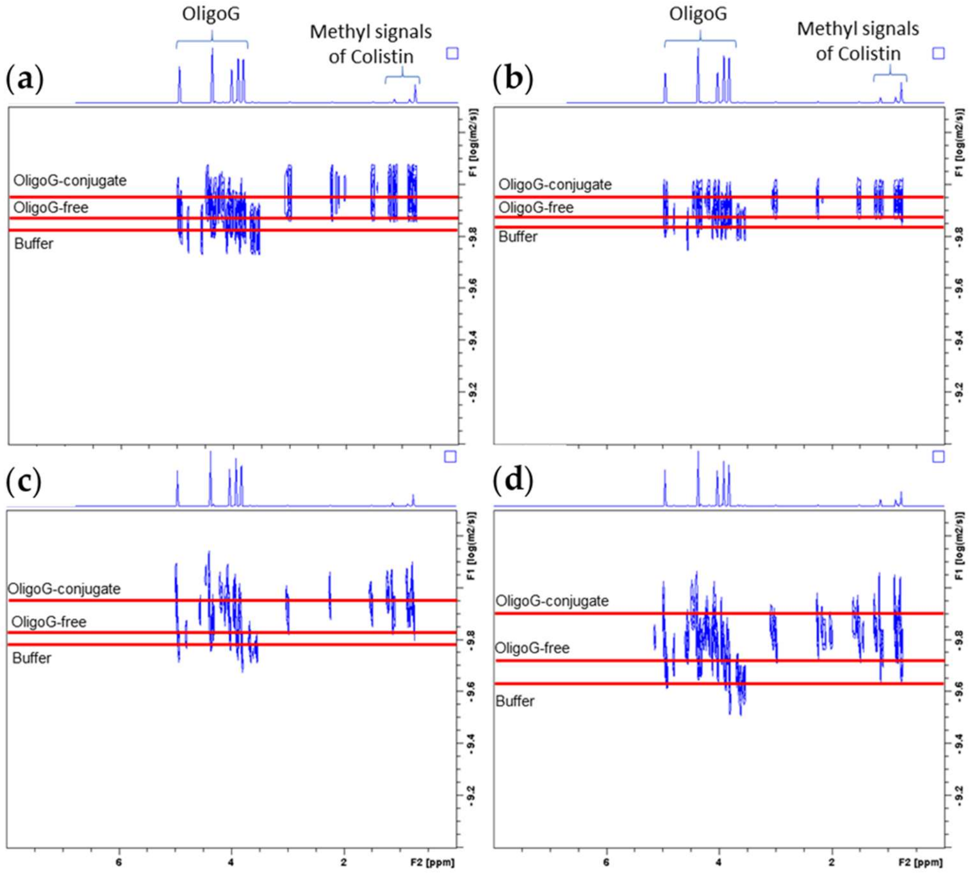

Figure S3. Diffusion-ordered spectroscopy (DOSY) of (**a-c**) three different batches of OligoG-A-colistin conjugates and (**d**) one batch of OligoG-E-colistin conjugate. The assignment of the unique signals for OligoG and colistin is indicated at the top of each panel and the red lines indicate the average diffusion coefficients of the molecules.

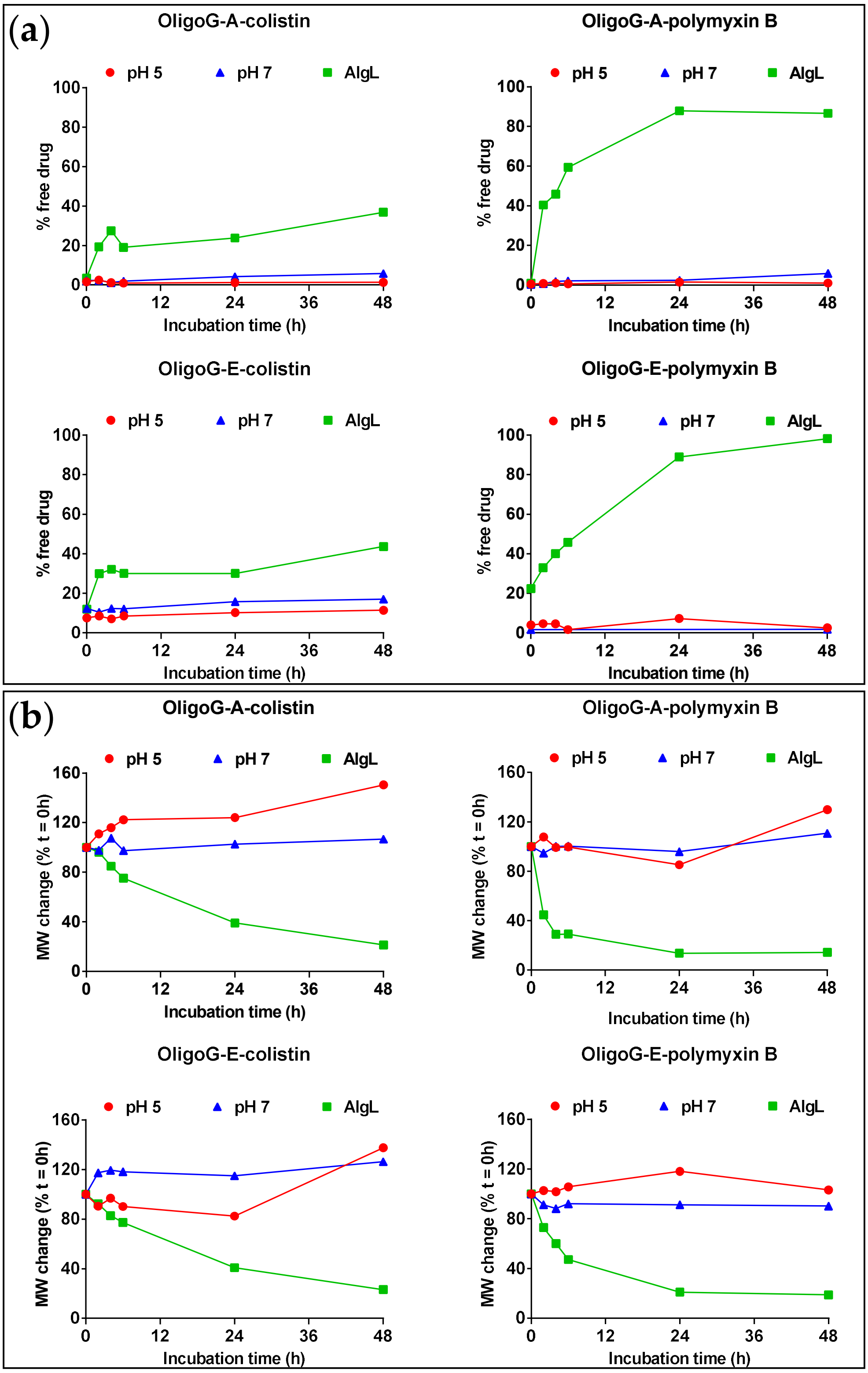

Figure S4. Drug release of OligoG-polymyxin conjugates in phosphate buffered saline (PBS) at pH 5, pH 7 or pH 7 containing alginate lyase (AlgL). (**a**) Content of free polymyxin and (**b**) change in molecular weight were determined by fast protein liquid chromatography (FPLC) and size exclusion chromatography with refractive index detection (SEC-RI), respectively, over 48 h incubation. Abbreviations: A, amide; E, ester.

Table S1. Gram-negative bacterial isolates used for characterisation of OligoG-polymyxin conjugates.

Bacterial Isolate	Genotype	Origin
P. aeruginosa R22	VIM-2	China
P. aeruginosa MDR 301	VIM-2	Poland
P. aeruginosa NH57388A		Denmark
P. aeruginosa NCTC 10662		Reference strain
K. pneumoniae KP05 506	NDM-1	India
K. pneumoniae IR25	NDM-1	India
A. baumannii MDR ACB	MDR	Libya
A. baumannii 7789	MDR	United Kingdom
E. coli AIM-1	AIM-1	Australia
E. coli IR57	NDM-1	India
E. coli 5702		United Kingdom
E. coli NCTC 10418		Reference strain
E. coli PN21	*mcr-1*	Thailand
E. coli PN25	*mcr-1*	Thailand
E. coli PN26		Thailand
E. coli ATCC 25922		Reference strain

VIM-2, carbapenem-hydrolyzing metallo-β-lactamase; NDM-1, New Delhi metallo-β-lactamase; AIM-1, metallo-β-lactamase; MDR, multidrug resistant; *mcr-1*, plasmid-encoded colistin resistance.

Table S2. Physicochemical characteristics and batch details of OligoG-polymyxin conjugates used in this study.

Tested Compound	Mw (g/mol) (PDI) by SEC-RI*	Mw (g/mol) (PDI) by SEC-MALS	Drug Content (% *w/w*)	Molar Ratio (per Colistin)	Conjugated NH_2 per Molecule	Free Drug (%)	Experiments Performed					
							Physicochemical	*Cytotoxicity*	*Antimicrobial Activity*	*Growth Curves*	*Biofilm Formation*	*PK-PD*
OligoG-A-colistin	25,500 (2.6)	12,300 (1.4)	8.7	4.6	2.8	5.7	x	x	x	x		
OligoG-A-colistin	22,500 (2.3)	9100 (1.3)	8.8	4.6	2.7	1.5	x		x	x	x	
OligoG-A-colistin	24,500 (2.3)	8300 (1.3)	9.1	4.4	3.6	2.5	x		x			
OligoG-A-colistin	27,000 (2.5)	8200 (1.2)	12.5	3.1	3.3	2.4	x		x			
OligoG-A-colistin	24,500 (2.2)	8200 (1.3)	8.1	5.0	4.6	4.0	x					x
OligoG-E-colistin	14,500 (2.3)	5900 (1.2)	12.9	3.0	N/A	2.0	x	x	x	x		
OligoG-E-colistin	16,500 (2.2)	5200 (1.3)	11.5	3.4	N/A	3.5	x		x	x	x	
OligoG-E-colistin	20,000 (3.1)	ND	8.3	4.9	N/A	2.7	x					x
OligoG-A-polymyxin B	23,000 (2.7)	12,800 (1.5)	8.0	5.1	1.9	1.6	x	x	x	x		
OligoG-A-polymyxin B	23,500 (2.2)	9100 (1.3)	6.1	6.8	2.0	1.6	x		x	x		
OligoG-E-polymyxin B	15,500 (2.1)	6200 (1.2)	7.0	5.9	N/A	2.7	x	x	x	x		

Abbreviations: A, amide; E, ester; SEC-MALS, size exclusion chromatography with multi-angle light scattering detection; SEC-RI, size exclusion chromatography with refractive index detection; PDI, polydispersity index; ND, not determined; N/A, not applicable. *Molecular weight was estimated relative to pullulan standards.

Table S3. Weight and number average molecular weights of OligoG and OligoG-colistin conjugates.

Tested Compound	Drug Content (% *w/w*)	Mn (kDa)	Mw (kDa)	Molar Ratio (per Colistin)*	Mn (kDa)*	Mw (kDa)*
OligoG	N/A	3.9	5.5	N/A	N/A	N/A
OligoG-A-colistin	9.1	6.6	8.3	2	4.3	8.3
OligoG-A-colistin	12.5	6.6	8.2	1.4	4.4	9.4
OligoG-A-colistin	8.1	6.4	8.2	2.5	4.2	8.0
OligoG-E-colistin	11.5	4.0	5.2	1.7	4.3	9.1

*Expected values assuming 100% conjugation calculated from the Mn and Mw values of OligoG and the % *w/w* of each sample.
Abbreviations: A, amide; E, ester; N/A, not applicable.

Table S4. Selectivity index (SI) values of OligoG-polymyxin conjugates against a range of Gram-negative bacterial pathogens.

Isolate	SI Value					
	**Colistin Sulphate**	**Polymyxin B**	**OligoG-E-Colistin**	**OligoG-E-Polymyxin B**	**OligoG-A-Colistin**	**OligoG-A-Polymyxin B**
P. aeruginosa R22	52	44	57	128	121	75
P. aeruginosa MDR 301	52	22	114	64	242	150
P. aeruginosa NH57388A	104	44	228	128	484	299
P. aeruginosa NCTC 10662	208	175	228	128	242	75
K. pneumoniae KP05 506	208	88	456	128	1936	598
K. pneumoniae IR25	413	88	456	256	242	75
A. baumannii MDR ACB	52	88	228	508	242	150
A. baumannii 7789	104	88	456	64	1936	598
E. coli AIM-1	208	22	228	64	1936	150
E. coli IR57	839	175	1839	508	3841	1196
E. coli 5702	208	44	114	128	968	75
E. coli NCTC 10418	3	1	4	4	8	9
E. coli PN21	3	3	7	8	8	9
E. coli PN25	208	88	228	128	484	598
E. coli PN26	104	22	57	64	242	19
E. coli ATCC 25922	52	44	57	128	121	75
Mean	**184**	**66**	**313**	**154**	**862**	**272**

Selectivity index (SI) = IC_{50} (µg/mL)/MIC (µg/mL). Mean SI is shown in **bold**. Abbreviations: A, amide; E, ester.

Table S5. Microbiological efficacy (MICs) of OligoG-colistin conjugates in the presence of alginate lyase in Mueller–Hinton (MH) broth or after pre-incubation with alginate lyase against Gram-negative bacterial pathogens.

Isolate	Tested Compound MIC (µg/mL Drug Base) at Indicated Alginate Lyase Concentration (U/mL)								
	**OligoG-A-Colistin**			**OligoG-E-Colistin**			**Colistin Sulphate**		
	**0**	**1**	**10**	**0**	**1**	**10**	**0**	**1**	**10**
	**Alginate Lyase in MH Broth**								
P. aeruginosa MDR 301	1	1	0.25	0.5	1	0.25	0.063	0.063	0.25
K. pneumoniae KP05 506	0.063	0.125	0.063	0.125	0.25	0.063	0.008	0.063	0.008
E. coli AIM-1	0.00002	0.0001	0.0002	0.000008	0.0002	0.0002	0.00006	0.00006	0.0002
A. baumannii 7789	0.063	0.063	0.008	0.125	0.125	0.125	0.002	0.004	0.004
	**Pre-incubation with Alginate Lyase**								
P. aeruginosa MDR 301	1	0.25	0.25	0.5	0.125	0.125	0.063	0.063	0.125
K. pneumoniae KP05 506	0.063	0.008	0.004	0.125	0.063	0.063	0.008	0.004	0.004
E. coli AIM-1	0.00002	0.001	0.0002	0.000008	0.001	0.001	0.00006	0.001	0.0002
A. baumannii 7789	0.063	0.016	0.004	0.125	0.063	0.063	0.002	0.002	0.002

Abbreviations: A, amide; E, ester.

Table S6. Microbiological efficacy (MICs) of polymyxins and antibiotic conjugates in the absence and presence of mucin against Gram-negative bacterial pathogens.

Tested Compound ↓ Mucin (%) →	MIC (µg/mL Drug Base)											
	***P. aeruginosa* R22**			***A. baumannii* MDR ACB**			***K. pneumoniae* IR25**			***E. coli* IR57**		
	**0**	**0.2**	**2**	**0**	**0.2**	**2**	**0**	**0.2**	**2**	**0**	**0.2**	**2**
OligoG-A-colistin	4	4	64	0.125	4	64	0.125	8	>128	0.063	2	8
OligoG-E-colistin	1	16	64	1	16	64	0.063	32	>128	0.063	4	64
0.2% OligoG + colistin	0.25	2	32	ND	ND	ND	ND	ND	ND	<0.063	2	4
2% OligoG + colistin	1	4	32	ND	ND	ND	ND	ND	ND	0.25	0.5	2
Colistin sulphate	0.5	2	16	0.5	1	16	0.063	1	64	0.125	1	2
OligoG-A-polymyxin B	2	64	>128	4	16	128	2	16	>128	2	16	32
OligoG-E-polymyxin B	0.5	2	32	0.25	1	16	0.125	2	128	0.125	0.5	1
0.2% OligoG + polymyxin B	0.5	4	64	ND	ND	ND	ND	ND	ND	0.125	2	4
2% OligoG + polymyxin B	1	4	32	ND	ND	ND	ND	ND	ND	<0.063	0.5	2
Polymyxin B	0.25	4	64	0.125	1	16	0.125	4	64	0.5	1	4

Abbreviations: A, amide; E, ester; ND, not determined.

Table S7. Comparison of the effect of growth medium (AS medium and MH broth) on antimicrobial activity (MIC determinations) of polymyxins and antibiotic conjugates.

Tested compound ↓ Medium →	MIC (μg/mL Drug Base)											
	P. aeruginosa **MDR 301**		***P. aeruginosa*** **NH57388A**		***K. pneumoniae*** **KP05 506**		***E. coli*** **IR57**		***E. coli*** **5702**		***A. baumannii*** **7789**	
	**AS**	**MH**	**AS**	**MH**	**AS**	**MH**	**AS**	**MH**	**AS**	**MH**	**AS**	**MH**
OligoG-A-colistin	4	1	2	0.5	2	0.125	0.125	0.125	0.25	0.063	2	0.125
OligoG-E-colistin	4	0.5	1	0.25	1	0.125	0.063	0.25	0.125	0.031	1	0.125
Colistin sulphate	2	0.5	1	0.25	1	0.125	0.125	0.125	0.125	0.031	1	0.25
OligoG-A-polymyxin B	16	2	4	1	8	0.5	0.5	2	1	0.25	4	0.5
OligoG-E-polymyxin B	8	0.5	2	0.25	2	0.25	0.125	0.5	0.25	0.063	1	0.5
Polymyxin B	4	0.5	1	0.25	1	0.125	0.125	0.5	0.25	0.063	0.5	0.125

Abbreviations: A, amide; E, ester.

Table S8. Fractional inhibitory concentration index (FICI) values of OligoG-colistin conjugates or colistin in combination with azithromycin dihydrate.

**Tested Compound**	***P. aeruginosa*** **MDR 301**	***K. pneumoniae*** **KP05 506**	***A. baumannii*** **7789**	***E. coli*** **NCTC 10418**
OligoG-A-colistin + Azithromycin	1.35 (Additive)	3.15 (Indifferent)	3.40 (Indifferent)	0.64 (Additive)
OligoG-E-colistin + Azithromycin	1.45 (Additive)	2.43 (Indifferent)	2.53 (Indifferent)	0.46 (Synergy)
Colistin sulphate + Azithromycin	1.51 (Additive)	2.20 (Indifferent)	1.14 (Additive)	0.83 (Additive)
